# Supplementary material for: Identification of photoperiod-induced specific miRNAs in the adrenal glands of Sunite sheep (Ovis aries)
Source: Front Vet Sci. 2022 Jul 22;9:888207. doi: 10.3389/fvets.2022.888207 (PMC9354845; doi:10.3389/fvets.2022.888207)
Supplement: Supplementary file 1 [file Data_Sheet_1.ZIP › Supplementary file/miRDeep2 file.zip!/miRDeep2 file/Know_Structure/SP42A3/oar-let-7a.pdf]

miRBase precursor : oar-let-7a
Total read count : 345801
oar-let-7a read count : 344033
remaining reads : 1768

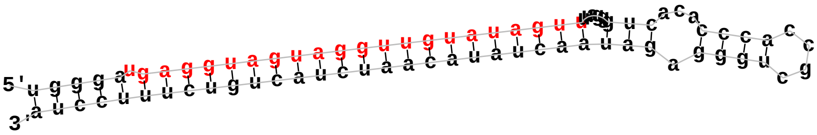

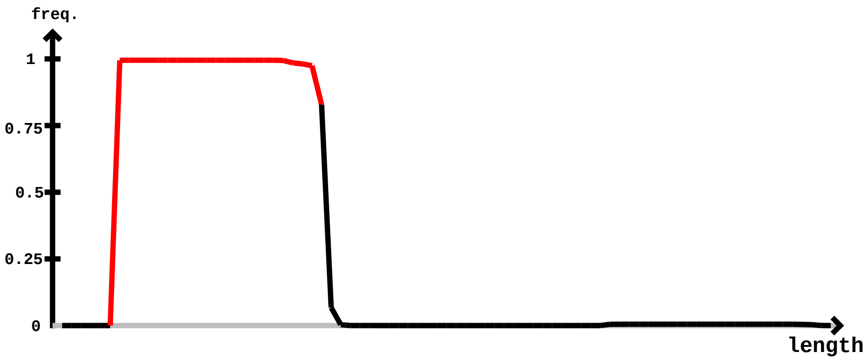


```
     oar-let-7a
5'- ugggaugagguaguagguuguauaguuuuagggucacacccaccgcugggagauaacuauacaaucuacugucuuuccua   -3'    exp
    (((((.(((((((((((((((((((((.....(((...((((....)))).)))))))))))))))))))))))))))))    reads      mm         sample
    ....augagguaguagguuguauag.......................................................    1          0          seq
    ....augagguaguagguuguauagu......................................................    6          0          seq
    ....Uugagguaguagguuguauagu......................................................    2          1          seq
    ....augagguaguagguuguauaguu.....................................................    16         0          seq
    ....Uugagguaguagguuguauaguu.....................................................    7          1          seq
    .....ugagguaguagguugua..........................................................    235        0          seq
    .....ugagguaguagguuUua..........................................................    2          1          seq
    .....ugagguaguaAguugua..........................................................    13         1          seq
    .....ugagguUguagguugua..........................................................    1          1          seq
    .....ugagguagGagguugua..........................................................    33         1          seq
    .....ugaAguaguagguugua..........................................................    1          1          seq
    .....ugagguUguagguuguau.........................................................    1          1          seq
    .....ugagguaguaAguuguau.........................................................    16         1          seq
    .....ugagguaguagguuUuau.........................................................    13         1          seq
    .....ugagguagGagguuguau.........................................................    262        1          seq
    .....ugaAguaguagguuguau.........................................................    16         1          seq
    .....ugagguaguagguuguau.........................................................    2830       0          seq
    .....ugagguagGagguuguaua........................................................    129        1          seq
    .....ugagguaguagguuguaua........................................................    1199       0          seq
    .....ugagguagGagguuguauag.......................................................    300        1          seq
    .....ugagguaguagguuguauag.......................................................    1928       0          seq
    .....ugagguaguagguuguauagu......................................................    44476      0          seq
    .....ugagguagGagguuguauagu......................................................    5914       1          seq
    .....ugagguaguagguuguauaguu.....................................................    242496     0          seq
    .....ugagguagGagguuguauaguu.....................................................    20720      1          seq
    .....ugagguaguagguuguauaguuu....................................................    22116      0          seq
    .....ugagguaguagguuguauaguuuA...................................................    189        1          seq
    .....ugagguaguagguuguauaguuuu...................................................    925        0          seq
    .....ugagguaguagguuguauaguuuua..................................................    12         0          seq
    .....ugagguaguagguuguauaguuuuaggg...............................................    1          0          seq
    .....ugagguaguagguuguauaguuuuagggu..............................................    1          0          seq
    ......gagguaguagguuguau.........................................................    1          0          seq
    ......gagguagGagguuguau.........................................................    1          1          seq
    ......gagguaguagguuguauag.......................................................    2          0          seq
    ......gagguagGagguuguauagu......................................................    1          1          seq
```

oar-let-7a

```
ugggaugagguaguagguuguauaguuuuagggucacacccaccgcugggagauaacuauacaaucuacugucuuuccua
......gagguaguagguuguauagu......................................................    16      0       seq
......gagguagGagguuguauaguu.....................................................    16      1       seq
......gagguaguagguuguauaguu.....................................................    121     0       seq
......gagguaguagguuguauaguuu....................................................    10      0       seq
.......agguaguagguuguauaguuu....................................................    1       0       seq
........gguaguagguuguauaguu.....................................................    1       0       seq
.........guaguagguuguauaguu.....................................................    1       0       seq
..........uaguagguuguauaguuu....................................................    2       0       seq
..........uaguagguuguauaguuuuagg................................................    1       0       seq
..........uaguagguuguauaguuuuaggg...............................................    1       0       seq
...........................uuagggucacacccaccgcug................................    1       0       seq
...........................uuagggucacacccaccgcuggg..............................    1       0       seq
...........................uuagggucacacccaccgcuggga.............................    11      0       seq
...........................uuagggucacacccaccgcugggagau..........................    1       0       seq
...................................................gauaacuauacaaucuacugucu......    1       0       seq
.....................................................uaacuauacaaucuacugucuuu....    3       0       seq
.....................................................uaacuauacaaucuacugucuuuc...    1       0       seq
......................................................aacuauacaaucuacugucuuuc...    1       0       seq
........................................................cuauacaaucuacugucu......    2       0       seq
........................................................Auauacaaucuacugucuu.....    1       1       seq
........................................................cuauacaaucuacugucuu.....    12      0       seq
........................................................cuauacaaucuacugucuuu....    256     0       seq
........................................................cuauacaaucuacuguUuuc...    1       1       seq
........................................................cuauacaaucuacugucuuuc...    243     0       seq
........................................................cuauacaaucuacugucuuucc..    1099    0       seq
........................................................cuauacaaucuacugucuuuccu.    54      0       seq
.........................................................uauacaaucuacugucuuuc...    1       0       seq
.........................................................uauacaaucuacugucuuucc..    12      0       seq
.........................................................uauacaaucuacugucuuuccu.    44      0       seq
..........................................................auacaaucuacugucuuucc..    1       0       seq
..........................................................auacaaucuacugucuuuccu.    18      0       seq
..........................................................auacaaucuacugucuuuccua    1       0       seq
```
